# Perceptions of care after end-of-treatment among younger women with different gynecologic cancer diagnoses – a qualitative analysis of written responses submitted via a survey

**DOI:** 10.1186/s12905-020-01133-z

**Published:** 2020-12-22

**Authors:** Elisabet Mattsson, Lisa Ljungman, Kim Einhorn, Inger Sundström Poromaa, Karin Stålberg, Anna Wikman

**Affiliations:** 1 Department of Health Care Sciences, Ersta Sköndal Bräcke University College, Stockholm, Sweden; 2 Department of Women’s and Children’s Health, Uppsala University, Uppsala, Sweden

**Keywords:** Young adult, Cancer, Gynecologic, Oncology, Survivorship, Quality of life

## Abstract

**Background:**

Less attention has been given to younger adults’ psycho-oncology care needs than to children and older adults with cancer. The aim was to explore how care following end-of-treatment was perceived by women treated for different gynecologic cancer diagnoses during younger adulthood.

**Methods:**

A sample of 207 women diagnosed with gynecologic cancer 2008 to 2016, aged 19–39 at time of diagnosis answered one open-ended question regarding important aspects of care after end-of-treatment. The written responses were analyzed with manifest content analysis and presented in relation to the women’s diagnoses, i.e., cervical (*n* = 130), ovarian (*n* = 57), and other gynecologic cancer diagnoses (*n* = 20).

**Results:**

The analysis resulted in three categories: *Unmet long-term supportive care needs*, *Satisfying long-term supportive care*, and *Health care organizational difficulties*. Over half of the women (66.7%) described unmet care needs. The corresponding figures were 80.7, 63.1 and 50% for women diagnosed with ovarian, cervical and other gynecologic cancer diagnoses, respectively. Satisfying supportive care were described by approximately one quarter of the women (26.1%). Among women diagnosed with ovarian cancer 14% described satisfying supportive care. The corresponding figures were 26.9 and 30% for women diagnosed with cervical cancer and other gynecological diagnoses, respectively. Approximately one quarter of the women, irrespectively of diagnosis, described aspects related to health care organizational difficulties (28%).

**Conclusions:**

The results highlight the importance of good quality care linked to the diagnosis and based on an understanding of the woman’s need, desire and expectation of support after end-of-treatment.

## Background

Young adults with cancer face specific challenges and needs that differ from those of children and older adults with cancer [1, 2] including delays in diagnosis [1, 3], difficulties with adherence to treatment [2, 3], financial concerns [1], and more pronounced psychological supportive care needs [1, 4]. Sexual problems pose great concerns, particularly among women [5], and the importance of counselling young adult cancer patients around fertility and sexuality issues has been stressed [5, 6]. Also, the 5-year survival rate of persons diagnosed with cancer between the ages of 15 and 29 has seen little improvement over the past decades, compared to younger or older cancer patients [7].

Gynecologic cancers, i.e., cervical, ovarian, uterine, vaginal and vulvar cancer, are the second most common cancers among women [8–11] and account for approximately 8% of all cancer diagnoses among women aged 20–39 years [8]. Women diagnosed with cervical cancer are often premenopausal [12], i.e., diagnosed before 50 years of age. Consequences of radiation treatment are common distressing symptoms, and infertility related to cancer treatment represents an important care need in this group [12]. Ovarian cancer is considered a low-prevalence but high-consequence disease [9]. It has the poorest prognosis of all gynecologic malignancies [9]. Following end-of-treatment, women diagnosed with ovarian cancer face regular surveillance including a high likelihood of recurrence [10]. Contrary to ovarian cancer, the prognosis for premenopausal women with early stage endometrial cancer is favorable, with a 5-year survival rate greater than 90% [11]. However, the standard surgical treatment, i.e., total abdominal hysterectomy, bilateral oophorectomy, and pelvic/para-aortic lymphadenectomy, significantly reduce the post-operative quality of life for young women due to symptoms of menopause, fertility loss, lymphedema and increased risk of cardiovascular disease [13]. Lastly, vulvar cancer can affect younger women with high-risk human papillomavirus infection [8]. Long-term impact of vulvar surgery often implies dyspareunia, fatigue, pain, and sexual problems [14]. Taken together, despite different disease trajectories for each gynecologic cancer diagnosis, the treatments may cause side-effects such as cardiovascular disease, fatigue, infertility, pain, sexual dysfunction, urinary complications, and premature menopause among younger women [13–15].

Women diagnosed with gynecologic cancer face not only physical symptoms of disease and treatment, but a multitude of psychological and social consequences. The emotional impact of gynecologic cancer is significant and clinical levels of anxiety, depression, and posttraumatic stress disorders (PTSD) as well as cancer-specific distress have been reported among survivors [16–19]. In addition, survivors report a gap between supportive care services and the need of such services [18, 19]. Supportive care needs in the context of cancer has been defined as “the provision of the necessary services for those living with or affected by cancer to meet their informational, emotional, spiritual, social, or physical needs during the diagnostic, treatment or follow up phases encompassing issues of health promotion and prevention, survivorship, palliation, and bereavement” [20]. The extent of unmet supportive care needs is, in turn, often associated with symptoms of anxiety, depression, PTSD and poorer quality of life [19]. In most previous studies, women diagnosed during young, middle, and late adulthood are lumped together and, consequently, no conclusions can be drawn as to whether these results can be generalized to women diagnosed at a younger age despite findings showing that younger age is a risk factor for greater unmet needs [21]. In fact, few studies in general have specifically focused on mental- and physical health of women diagnosed with cancer during young adulthood [22] with less attention given to young adults’ psycho-oncology care needs than to children and older adults with cancer [3]. Against this background, the aim of the present study was to explore how care following end-of-treatment was perceived by women treated for different gynecologic cancer diagnosis during younger adulthood.

## Methods

### Study design

This study was part of a cross-sectional survey using mixed-methods to explore important aspects of care following end-of-treatment perceived by women diagnosed with gynecologic cancer during young adulthood. The present study includes data from the qualitative part of the survey. Eligibility criteria were: a diagnosis of gynecologic cancer between 2008 and 2016 included in the Swedish Quality Registry for Gynecologic Cancer, aged 19–39 at time of diagnosis, and having completed primary treatment. Exclusion criteria were: borderline tumors of the ovary, or carcinoma in situ of the cervix, vulva, or vagina.

### Study sample

Ethical approval to conduct the study was granted by the Regional Ethical Review Board in Uppsala, Sweden (Reference number: 2016/221). Potential participants were identified via the Swedish Quality Register for Gynecologic Cancer [23]. The register consists of four sub-registries: i) ovarian cancer (ICD-10: C56.9, C57.0, C48.1, C48.2, C76.2, C76.3), including fallopian tube, peritoneal, and abdominal or pelvic cancers, available since 2008; ii) uterine cancer (ICD-10: C54), available since 2010; iii) cervical and vaginal cancer (ICD-10: C52, C53), since 2011; iv) vulvar cancer (ICD-10: C51), available since 2012. The data input in the register is validated against the Swedish Cancer register and medical records [23]. Information on contact details were obtained via data linkages to the SPAR-register (the Swedish personal address register).

### Data collection

Potential participants were sent information about the study together with a study-specific questionnaire to complete and return by post. The information letter contained a study code, which enabled participants to complete the survey online should they prefer. By responding to the survey, participants provided informed consent to participate in the study. Up to two reminder letters were sent. In brief, the questionnaire included questions addressing cancer-related distress, needs for psycho-social support, previous psychological/psychiatric distress, and received psychological support. In addition, socio-demographic information was collected and included marital status, number of children, and education. Answers were given via fixed options and/or written open responses. For a detailed presentation of results of the study-specific questionnaire, see Mattsson et al. (2018) [4]. The present study includes data from the qualitative part of the survey, not previously reported. For this part, one open-ended question was included  where women were asked to freely describe anything they felt was important to share in relation to their needs and experiences after end of treatment (“Please describe anything you feel is important to share in relation to your needs and experiences after end of treatment”).

Clinical data were obtained from the Swedish Quality Register for Gynecologic Cancer [23] and included date of birth, diagnosis, date of diagnosis, treatment, and date of treatment completion. As information on recurrence is not yet reliable in the register, such data were not obtained.

### Data analysis

The written responses were analyzed with content analysis, a method that can be used to draw valid conclusions about a manifest message by systematic identification of specified written characteristics [24]. The responses were read repeatedly by the first and last author to gain an overall understanding. Words and sentences, i.e., recording units, containing relevant information regarding important aspects of care following end-of-treatment were identified. The first and last author grouped recording units into exclusive categories reflecting central messages. Recording units in the same category are assumed to have a similar meaning, based on either the precise meaning of the words or of words sharing the same connotations. The authors defined the boundaries of each category and developed final descriptions of the central characteristics of each category. The analytic process was flexible and iterative, i.e., steps were repeated when needed and the analysis was reviewed in relation to the raw data during all steps. When categories were identified, the first and last author scrutinized the results and were involved in discussion until all authors felt the results adequately reflected the content in the written responses. Counting was integral to the analysis process to recognize potential patterns in data and deviations from those patterns, as well as to make analytic generalizations from data [25]. Consequently, recording units in the same category were counted (numbers (n) and percentages (%)) and presented in relation to the women’s diagnoses, i.e., cervical, ovarian, and other gynecologic cancer. Even if a respondent mentioned a certain recording unit several times, it was only counted once in the results. The COREQ (consolidated criteria for reporting qualitative research) checklist was used to guide the analysis and the reporting [26]. However, participants did not provide feedback on the findings as no relationship was established between the researchers and participants due to the study’s design, i.e., survey. Data were analyzed using NVivo Pro for Windows version 11.3 (QRS International Pty. Ltd., Australia).

Sample characteristics are described by frequencies and percentages, n (%), for categorical variables and by means and standard deviations (SD), supplemented by median and range, for continuous variables. The statistical analyses were performed using SPSS Statistics for Windows, version 24 (IBM Corp., Armonk, N.Y., USA).

## Results

Of 646 eligible, 337 (52%) women responded to the survey. Of these, 207 (61.4%) women responded to the specific open-ended question about what they felt important to tell us about regarding care following end-of-treatment. Characteristics of the study sample are shown in Table 1.

**Table 1:** Clinical and socio-demographic characteristics of 207 women after end-of-treatment for gynecologic cancer

Characteristic	Total (***n*** = 207)	Cervical cancer (***n*** = 130, 62.8%)	Ovarian cancer (***n*** = 57, 27.5%)	Other^**a**^ (***n*** = 20, 9.7%)
	Mean (SD)	Mean (SD)	Mean (SD)	Mean (SD)
Age at diagnosis	34.2 (4.9)	34.6 (4.1)	32.7 (6.2)	36.1 (4.7)
Age at time of study	37.1 (5.1)	37.2 (4.6)	36.4 (6.1)	39.1 (5.2)

	n (%)	n (%)	n (%)	n (%)
Treatment				
Surgery	110 (53.1)	86 (66.2)	11 (20.3)	13 (65.0)
Surgery and/or chemotherapy and/or radiotherapy	97 (46.9)	44 (33.8)	46 (79.7)	7 (35.0)
Time since diagnosis				
≤ 1 year	52 (25.1)	38 (29.2)	11 (19.3)	3 (15.0)
2–4 years	111 (53.6)	71 (54.6)	27 (47.4)	13 (65.0)
≥ 5 years	44 (21.3)	21 (16.2)	19 (33.3)	4 (20.0)
Time since end-of-treatment				
≤ 1 year	68 (32.8)	46 (35.4)	17 (29.8)	5 (25.0)
2–4 years	103 (49.8)	69 (53.1)	23 (40.4)	11 (55.0)
≥ 5 years	36 (17.4)	15 (11.5)	17 (29.8)	4 (20.0)
Cohabitation at time of study				
Cohabiting	146 (70.5)	96 (73.8)	38 (66.7)	12 (60.0)
Non-cohabiting	61 (29.5)	34 (26.2)	19 (33.3)	8 (40.0)
Education				
University degree	140 (67.6)	87 (66.9)	41 (71.9)	12 (60.0)
Nine-years compulsory/upper secondary	64 (30.9)	41 (31.5)	16 (28.1)	7 (35.0)
Missing	3 (1.5)	2 (1.6)	0 (0)	1 (5)
Children				
Yes	122 (58.9)	91 (70.0)	23 (40.4)	8 (40.0)
No	84 (40.6)	38 (29.2)	34 (59.6)	12 (60.0)
Missing	1 (0.5)	1 (0.8)	0 (0)	(0)

^a^Other = Endometrial (*n* = 16), pelvic (*n* = 1), and vulvar cancer (*n* = 3); *n* numbers, *SD* Standard deviation

The median (range) age at diagnosis for the total sample (*n* = 207) was 35 (21–41) years. The corresponding figures for women diagnosed with cervical cancer (*n* = 130) were 35 (25–41) years, and 34 (21–41) and 38 (23–41) years for women diagnosed with ovarian cancer (*n* = 57) and other diagnoses (*n* = 20), i.e., endometrial, pelvic and vulvar cancer, respectively. Most of the respondents (67.6%) had a university degree. The median (range) age at time of study for the total sample (*n* = 207) was 37 (25–46) years. For most women (*n* = 111, 53.6%) time since diagnosis ranged between 2 and 4 years. All participants had completed treatment at the time of the survey. Nearly half received multimodal treatment (*n* = 97, 46.9%). Notably, the proportion of multimodal treatment among women with ovarian cancer (*n* = 46, 79.7%) was more than double that of the proportion of multimodal treatment among women with cervical (*n* = 44, 33.8%) or other gynecologic cancers (*n* = 7, 35.0%). Similarly, the proportion of women with ovarian cancer (*n* = 23, 40.4%) who reported having children was lower than among women with cervical cancer (*n* = 91, 70.0%) but like other gynecologic cancers (*n* = 8, 40.0%).

The content analysis resulted in three categories described below: *Unmet long-term supportive care needs, Satisfying long-term supportive care*, and *Health care organizational difficulties*. See Table 2 for a presentation of citations and numbers of recording units (n, %) in each category related to the different gynecological cancer diagnoses.

**Table 2:** A presentation of categories, examples of citations and numbers of recording units in each category

Category	Example, citations	All women (***n*** = 207)	Women diagnosed with cervical cancer (***n*** = 130)	Women diagnosed with ovarian cancer (***n*** = 57)	Women diagnosed with other gynecologic cancers^**a**^ (***n*** = 20)
		*Recording units* *n (%)*	*Recording units* *n (%)*	*Recording units* *n (%)*	*Recording units* *n (%)*
*Unmet long-term supportive care needs*	*”Everyone ignores the child/infertility viewpoint, it’s not so fun to go from what I at least thought fertile to infertile. Psychologically this is a trauma, but it feels like no one wants to understand.”* *“I think there’s a lack of a more holistic view of people in healthcare. The body is treated with such finesse, but the mental parts are left aside.”* *“A lack of information I needed. In my case hysterectomy and I was shocked by the problems after the operation and regretted it immediately and still do.”*	138 (66.7)	82 (63.1)	46 (80.7)	10 (50.0)
*Satisfying long-term supportive care*	*”About 8 months after end-of-treatment I participated in a cancer rehabilitation group. We were a group that met about twice a week. Once a week we trained with a physiotherapist and once a week we had group sessions with counsellors. I thought this was incredibly good.”* *“It has been very nice to have the same doctor during all my follow-up appointments. I’ve also been able to reach her* via *telephone when I have been wondering about things. Very good to meet a counsellor, someone external, to talk with – until I felt I was “done” with the trauma.”*	54 (26.1)	35 (26.9)	8 (14.0)	7 (35.0)
*Health care organizational difficulties*	*”I had 4–5 doctors during a one-year period and it felt very insecure and impersonal.”* *“Help in hurrying up the healthcare would be needed. It shouldn’t take four weeks to get answers from a scan when they say it should take two weeks.”*	58 (28.0)	39 (30.0)	14 (24.6)	5 (25.0)

^**a**^Other = Endometrial (*n* = 16), pelvic (*n* = 1), and vulvar cancer (*n* = 3)

### Unmet long-term supportive care needs

Over half of the women who responded to the question described aspects belonging to the category *Unmet long-term supportive care needs* (66.7%). A majority of women diagnosed with ovarian cancer described unmet needs (80.7%). The corresponding figures were 63.1 and 50% for women diagnosed with cervical cancer and other gynecologic cancer diagnoses, respectively. The content of the category includes descriptions of unmet psychological care needs after end-of-treatment, sometimes perceived as neglected by health care professionals. Some women considered information regarding late effects from surgery and treatments inadequate. In addition, women described distress regarding sexuality and infertility issues that was not acknowledged by the health care team. Concerns regarding how to deal with sexual problems and how these problems affect a relationship were raised. Some women also described a need for follow-up care where partners were offered to participate actively.

### Satisfying long-term supportive care

About one in four women described aspects belonging to the category *Satisfying long-term supportive care* (26.1%). However, among women diagnosed with ovarian cancer 14% described satisfying supportive care. The corresponding figures were 26.9 and 30% for women diagnosed with cervical cancer and other gynecologic cancer diagnoses, respectively.

The content of the category includes descriptions of satisfaction with the given care. These women stressed the importance of long-term continuity of health care professionals and the routinely offered psychosocial support following end-of-treatment. Other factors described as important were easy access to health care professionals and the availability of organized group support. Key concepts of good care were described by the women as meeting an interdisciplinary health care team involved in long-term health care management and psychosocial support. Meeting other women diagnosed with cancer together with different representatives from the health care was also considered important.

### Health care organizational difficulties

Approximately one quarter of the women, irrespectively of diagnosis, described aspects belonging to the category *Health care organizational difficulties*.

The content of the category includes descriptions of experiences of non-continuity in the health care and worries related to extensive waiting times for test results. These aspects were in turned described to create worries. In addition, administration differences between health care regions regarding sick leave were described.

## Discussion

The aim of this study was to describe important aspects of gynecologic cancer care following end-of-treatment as perceived by younger adult women, and to explore these aspects in relation to the different gynecologic cancer diagnoses. The results revealed three categories representing the answers from the participants: *Unmet long-term supportive care needs*, *Satisfying long-term supportive care*, and *Health care organizational difficulties*.

The most prominent reported aspect of care belonged to the category *Unmet long-term supportive care needs* (66.7%). The proportion of women reporting answers in this category varied between the different gynecologic cancer diagnoses with the highest frequency observed among women with ovarian cancer (80.7%). Results from a large survey of supportive care needs including 303 women with gynecologic cancer found no association between reported needs and type of cancer [27]. However, one recent review of supportive care needs in cancer found associations with sociodemographic (e.g., younger age, lower socioeconomic status, having no children) and clinical factors (e.g., more advanced disease, multiple cancer sites) that influenced the magnitude of unmet needs [28]. Overall, women experienced that their psychological care needs were neglected by health care professional, and when treatment was completed, they were left alone with their feelings related to the cancer experience. Some also reported the period after end-of-treatment as particularly challenging. These findings correspond with previous studies of supportive care needs, where women described needs for distress screening and support [29, 30] and a desire to be offered appropriate supportive care services and follow-up after treatment [29–33]. A key barrier to psychosocial care perceived by patients is the perception by health care providers that psychosocial care is not needed [34].

Women also described a lack of information regarding late effects from the treatment they had received. Common important needs for information in general [35], or information about coping with e.g., fear of recurrence [35], side effects [35, 36] and daily living [36] have been reported previously. Not surprisingly, women described significant distress regarding sexuality and fertility. It is well known that women with gynecologic cancer may face additional, specific problems, compared with other cancer diagnoses, including loss of fertility, sexual dysfunction, bowel dysfunction, fecal and urinary incontinence and emotional and psychological issues related to body image, sexuality and relationships [9, 12, 13, 15]. In a longitudinal study of women with ovarian cancer, unmet needs in the domains of information, patient care and sexuality were reported to decrease over a two-year period following diagnosis, whereas needs relating to psychological and physical domains remained moderate-to-high during the same time period [37]. In contrast, in the present study unmet information needs, distress regarding sexuality and fertility were reported in the longer term, most women were at least two years post-diagnosis and comprised a much younger age group in general. This is line with previous studies that have shown younger women with gynecologic cancer to be at greater risk of distress and experiencing unmet needs [21].

One in four women reported answers belonging to the category *Satisfying long-term supportive care*. Answers in this category concerned continuity in terms of health care professionals during the whole disease trajectory, having been offered psychosocial support services routinely, and meeting other women in the same situation. These answers reflect previous findings regarding important factors with regard to support in relation to a cancer experience. A qualitative study exploring psychosocial distress, coping and social support among women with ovarian cancer reported that when the participants were asked who they thought would be most beneficial to talk to about their experiences of cancer, most women responded that another survivor would be the best [38]. Peer support and participation in other forms of support groups are associated with positive results and experiences among patients with gynecologic cancer [39]. It is important to note though, that the proportion of women who experienced satisfying long-term supportive care varied between the different gynecologic cancer diagnoses included in this study with the lowest figure observed among women with ovarian cancer. Women with ovarian cancer were treated with multimodal treatment to a greater extent than were the other diagnoses, which may indicate a more difficult treatment experience. In addition, ovarian cancer is associated with the poorest prognosis of all gynecologic cancers [9].

Health care organizational difficulties were described by approximately one quarter of the women in this study. These findings are in line with observations from the general cancer literature of limited service availability and accessibility concerning supportive care [34]. Other health care systems-based challenges have been reported, where women described difficulties with e.g., waiting times and scheduling. A desire for consistency and continuity in the health care organization was described in order to improve patients’ experiences of care [40]. Results from a recent Australian survey of supportive care needs found health service and information needs to be most prevalent, representing eight of the top ten reported supportive care needs, with the highest ranked need ‘being informed about your test results as soon as feasible’ [27].

### Study limitations

Although data analyzed were of a qualitative nature, participants were not interviewed but provided written answers to one specific question on needs and experiences after end of treatment, which precluded any further exploration of responses. On the other hand, it could be argued that the results truly represent what young women with gynecologic cancer consider important aspects of care following end-of-treatment, as the answers were not influenced by an interviewer. However, it must be noted that whether these results are representative of the experiences of women with gynecologic cancers of all ages, younger and older, cannot be established. Also, whether prior physical or psychological issues influenced these perceptions in any way is not known. Notably, our intention with the present study was not to provide generalizable conclusions, but rather to gain deeper understandings and generate hypotheses for future studies.

An important strength of the current study is the large number of participants included. This allowed a quantitative presentation of data in addition to the content analyses, which can be important in generating new hypotheses [25]. The different pattern of important aspects of care described between the different gynecologic cancers needs to be considered when interpreting the results. Results indicate that follow-up programs for young women suffering from gynecologic cancer need to consider the impact of the specific diagnosis on the disease trajectory. An important aspect for future research within psycho-oncology care is to evaluate how such programs best meet women’s multidimensional needs. However, whether different treatment regimens, the psychological prerequisites of the types of cancer, or some other aspect of the disease influence needs warrant further exploration.

### Clinical implications

Patterns of important aspects of care varied between the different diagnoses where women treated for ovarian cancer described unmet supportive care needs to a greater extent. At present, national guidelines are in place regarding follow-up care for women with gynecological cancer including being assigned a contact nurse through diagnosis, treatment and follow-up. However, there are regional variations and still, much focus remains on physical and medical issues during follow-up. Based on these results, we believe follow-up care after gynecological cancer must incorporate a broader view of support needs. Further studies exploring these patterns may be warranted, to develop more specific recommendations for psycho-oncology care and supportive care after gynecologic cancer.

## Conclusion

In conclusion, results point to the importance of good quality care linked to the diagnosis and based on an understanding of a person’s need, desire and expectation of support.

## Data Availability

Data sharing is not applicable to this article due to the ethically sensitive nature of the materials for which the authors do not have ethical permission to distribute. However, a de-identified dataset can potentially be made available by the corresponding author upon reasonable request, including an appropriate ethical approval.

## Abbreviations

COREQ: Consolidated criteria for reporting qualitative research
ICD: International Statistical Classification of Diseases
N: numbers
PTSD: Posttraumatic Stress Disorder
SD: Standard Deviation
SPAR: Swedish personal address register
